# Prepectoral Breast Reconstruction with Polyurethane-Coated Implants: A 117-Patient Single-Center Study with Objective Cosmetic Evaluation

**DOI:** 10.1007/s00266-026-05901-4

**Published:** 2026-05-18

**Authors:** Sevgi Kurt, Isil Akgun Demir, Naci Celik, Memet Yazar, Enver Ozkurt, Ertan Koc, Tomris Duymaz, Vahit Ozmen

**Affiliations:** 1 Department of Plastic, Reconstructive and Aesthetic Surgery, Istanbul Atlas University, Istanbul, Turkey; 2 Department of Plastic, Reconstructive and Aesthetic Surgery, Uskudar University, Istanbul, Turkey; 3 Department of General Surgery, Istanbul Florence Nightingale Hospital, Istanbul, Turkey; 4 Academy of Statistics (Private Practice), Istanbul, Turkey; 5 Department of Physiotherapy, Istanbul Bilgi University, Istanbul, Turkey; 6 Department of Histology and Embryology, Medipol University, Istanbul, Turkey

**Keywords:** Prepectoral breast reconstruction, Polyurethane-coated implants, Microthane implants, BCCT.core, Aesthetic outcomes, Complications

## Abstract

**Background:**

Prepectoral breast reconstruction with polyurethane (PU)-coated implants has been proposed to enhance implant stability and reduce complications. Evidence regarding their clinical and aesthetic performance remains limited.

**Methods:**

A retrospective review was performed on 117 patients (186 breasts) who underwent immediate prepectoral reconstruction with PU-coated implants between 2020 and 2025. Complications were analyzed with univariate and multivariable regression. Aesthetic outcomes were evaluated using BCCT.core software.

**Results:**

At a median follow-up of 15 months, 34 patients (29.1%) experienced at least one complication, most commonly rippling or upper-pole visibility (16.2%). Implant loss occurred in 3.4% and capsular contracture in 1.7%. Higher BMI, larger implant size, and skin-reducing mastectomy were associated with increased complication risk, although none remained independently significant. Chemotherapy had no measurable impact, and radiotherapy showed only a mild trend toward poorer aesthetic scores. Overall, 81.2% of patients achieved excellent or good BCCT.core results. Younger age and absence of complications were the strongest predictors of favorable aesthetic outcomes.

**Conclusions:**

PU-coated implants provided stable outcomes in prepectoral reconstruction, with low implant loss and rare contracture. Most patients achieved favorable cosmetic results without the need for additional reinforcement materials. Higher BMI, larger implants, smoking, and skin-reducing mastectomy increased complication risk primarily in univariate analyses. Prospective multicenter studies with longer follow-up and patient-reported outcomes are required to validate these findings.

**Level of Evidence III:**

This journal requires that authors assign a level of evidence to each article. For a full description of these Evidence-Based Medicine ratings, please refer to the Table of Contents or the online Instructions to Authors  www.springer.com/00266.

## Introduction

Implant-based breast reconstruction is the most common method following mastectomy, accounting for nearly 80% of reconstructions in the USA [1, 2]. Subpectoral placement was long considered the standard because the pectoralis major was believed to protect the implant and reduce flap-related complications [3]. However, it is associated with drawbacks such as animation deformity, chronic pain, muscle spasm, and limited lower-pole expansion [4, 5].

Better mastectomy techniques and more reliable flap assessment have renewed interest in prepectoral placement [6]. By avoiding muscle dissection, the prepectoral approach reduces postoperative pain, accelerates recovery, and eliminates animation deformity, and several reports demonstrate that its complication rates can be comparable to subpectoral reconstruction in appropriately selected patients [7, 8]. Concerns still include implant visibility, rippling [9], and possibly higher capsular contracture rates, especially in irradiated patients [10].

Polyurethane (PU)-coated implants were introduced to mitigate some of these issues. Their microporous foam surface promotes fibrovascular integration and stronger tissue adhesion, reducing the likelihood of malposition or capsular contracture [11]. This ‘Velcro effect’ is particularly relevant in the prepectoral plane, where stability relies entirely on the mastectomy flap. Recent clinical reports indicate that PU implants maintain stable position and deliver satisfactory contour in both prophylactic and oncologic cases [12, 13].

The aim of this study was to evaluate the clinical and aesthetic outcomes of direct-to-implant prepectoral breast reconstruction using PU-coated implants. We assessed complication rates, identified associated risk factors, and analyzed cosmetic results with an objective software-based method. The impact of adjuvant treatments, including radiotherapy and chemotherapy, was also examined. To our knowledge, this represents one of the larger single-center series focused exclusively on PU-based prepectoral reconstruction and provides practical insight into their performance in routine clinical practice.

## Methods

### Study Design and Patient Population

Between 2020 and 2025, we included patients who underwent nipple-sparing mastectomy (NSM) or skin-reducing mastectomy with free nipple–areola grafting (SRM-FNG), followed by immediate direct-to-implant prepectoral reconstruction using polyurethane-coated implants (Microthane Sublime Line®, Polytech Health and Aesthetics, Dieburg, Germany), including both Opticon and Replicon base designs selected according to breast dimensions and reconstructive requirements. Both therapeutic and prophylactic mastectomies were eligible. Unilateral reconstructions were accepted, and contralateral symmetrization procedures were planned when needed. Breast reduction or mastopexy on the contralateral side was performed only after the reconstructed breast had stabilized.

Patients were excluded if the nipple–areola complex required oncologic removal, flap viability was judged inadequate for direct-to-implant placement, or a two-stage expander-based approach was chosen. Smokers, patients with elevated BMI and those expected to receive radiotherapy were not excluded but were counseled about their increased risk of complications. SRM-FNG cases were included because the NAC was preserved and grafted for perfusion- or ptosis-related indications rather than for oncologic reasons. All patients provided written informed consent, including information about the rare but recognized risk of breast implant-associated anaplastic large-cell lymphoma (BIA-ALCL). All mastectomies were performed by the same breast surgeon and all reconstructions by the same plastic surgeon.

### Data Collection

Demographic and clinical variables were recorded, including age, BMI, smoking status, comorbidities, laterality, surgical indication, implant base design, and details of adjuvant treatments (radiotherapy and chemotherapy).

In patients undergoing mastectomy for node-positive disease, hypofractionated radiotherapy was administered at a total dose of 42.56 Gy delivered in 16 fractions. The radiation fields included the chest wall, axilla, and supraclavicular regions.

Genetic predisposition—BRCA1, BRCA2, and other pathogenic variants—was also documented.

### Complication Assessment

Postoperative complications were evaluated at each follow-up visit. Complications were classified into predefined categories: implant loss, flap necrosis, hematoma, seroma, infection, capsular contracture, implant malposition or rotation, and upper-pole contour changes such as rippling. Clinical examination was the primary assessment method. Imaging was obtained only when clinical findings were inconclusive.

### Aesthetic Evaluation

Standardized frontal photographs were taken preoperatively and during follow-up at 1, 3, and 6 months, and then annually. Aesthetic outcomes were evaluated using the Breast Cancer Conservative Treatment core (BCCT.core) software, an image-analysis tool originally developed for cosmetic assessment after breast-conserving surgery [14, 15]. For this study, scoring was performed only on photographs obtained after at least six postoperative months. In most cases, evaluation was based on images taken after the first postoperative year. All photographs were analyzed using a consistent protocol.

For each patient, the frontal photograph was uploaded into the software, and a four-level categorical score was generated (excellent, good, fair, poor) based on geometric indices of symmetry, contour, nipple position, and scar distribution. The system uses calculated indices rather than subjective grading; therefore, it offers a uniform and reproducible assessment of symmetry, contour, nipple position, and scar distribution that are also relevant in implant-based reconstruction; however, BCCT.core does not assess ptosis or patient-reported satisfaction and should therefore be interpreted as an objective surrogate of aesthetic outcome rather than a comprehensive cosmetic evaluation. In unilateral reconstructions, BCCT.core scores were interpreted with awareness of the inherent asymmetry relative to the native breast. Contralateral symmetrization procedures were planned only after stabilization of the reconstructed breast and were not routinely completed at the time of aesthetic evaluation; in addition, some patients did not wish to undergo further contralateral surgery. Therefore, BCCT.core results in unilateral cases reflect early postoperative aesthetic balance rather than final bilateral symmetry.

Age, BMI, and implant volume were examined across BCCT.core categories to explore their potential influence on cosmetic outcome. Binary logistic regression (good/excellent vs fair/poor) and ordinal logistic regression (excellent→poor) were used to explore predictors of cosmetic outcome.

### Surgical Technique

All NSM procedures and axillary dissections (SLND or ALND) were performed by the same breast surgeon, and all reconstructions by the same plastic surgeon. Skin incisions were selected according to breast size and degree of ptosis. Lateral or horizontal incisions were used for small or moderate breasts, whereas a Wise-pattern incision with NAC grafting was preferred in larger or more ptotic cases. In unilateral operations, incision planning aimed to maintain symmetry with the contralateral breast.

After mastectomy, flap viability was assessed clinically, with attention to thickness and perfusion. Thin or marginal flaps can reduce effective adhesion on the PU-coated surface. Anatomically shaped PU implants were placed directly in the prepectoral plane without ADM, mesh, or pocket-shaping sutures; neither ADM nor smooth implants were used. Our center has used PU-coated devices exclusively for the past five years, based on their positional stability and the lack of need for additional reinforcement. Pocket dissection was kept minimal to preserve contact between the implant and the overlying flap.

In patients with a submammary incision, the inferior mastectomy flap was anchored to the chest wall to limit incision migration and support the lower pole. Implants with moderate or high projection were chosen to improve contour and reduce rippling. Positioning was checked in the upright position to ensure full base contact and an appropriate shape; this also allowed small adjustments before adhesion developed. Once placed, the polyurethane surface adheres rapidly, limiting micro-movement and reducing the need for fixation sutures.

In NAC grafting cases, implant position was centered relative to the planned nipple site to avoid secondary adjustments after adhesion. Closed-suction drains were kept until the output was below 30 mL for two consecutive days. Perioperative intravenous antibiotic prophylaxis was administered at induction, followed by oral antibiotics continued until drain removal. This approach was adopted after earlier cases of premature drain removal and early seroma formation and was used to minimize the risk of infection in prepectoral direct-to-implant reconstruction. Patients wore a soft, non-compressive bra for 4–6 weeks.

### Statistical Analysis

Continuous variables were reported as mean ± standard deviation or median with range, depending on distribution. Categorical variables were presented as counts and percentages. Normality was assessed using the Shapiro–Wilk test. Group comparisons used the Student’s t test or the Mann–Whitney U test for continuous variables, and the chi-square test or Fisher’s exact test for categorical variables. BCCT.core categories were compared using the Kruskal–Wallis test.

Binary logistic regression was used to identify predictors of postoperative complications. Clinically relevant variables and those with *p* < 0.10 in univariate analysis were included in the multivariable model. Because the number of events was limited, the multivariable analysis was considered exploratory due to the low events-per-variable ratio.

Ordinal logistic regression (excellent→poor) and binary logistic regression (good/excellent vs fair/poor) were used to assess predictors of aesthetic outcome. Regression coefficients were exponentiated and presented as odds ratios (OR) with 95% confidence intervals (CI). Goodness of fit was assessed using standard metrics, including the Hosmer–Lemeshow statistic and pseudo-R^2^ indices (Cox–Snell, Nagelkerke, McFadden). A *p* value < 0.05 was considered statistically significant.

All analyses were performed in Python 3.11 (pandas, SciPy, statsmodels).

## Results

### Patient Characteristics

The mean patient age was 44.5 ± 9.0 years (median 43, range 27–71), with a mean BMI of 24.6 ± 3.6 kg/m^2^ (median 24.2, range 18.4–37.1). Mean implant volume was 391.9 ± 79.9 mL (median 390, range 185–560). Drains were removed after a mean of 14.6 ± 5.6 days. Median follow-up was 19 months (range 6–58) (Table 1).

**Table 1:** Baseline patient characteristics (N = 117)

Variable	Mean ± SD	Median (range)
Age (years)	44.5 ± 9.0	43 (27–71)
BMI (kg/m^2^)	24.6 ± 3.6	24.2 (18.4–37.1)
Implant volume (mL)	391.9 ± 79.9	390 (185–560)
Drain removal (days)	14.6 ± 5.6	14 (5–30)
Follow-up (months)	19.1 ± 10.0	19 (6–58)

Continuous variables are presented as mean ± SD and median (range)

*SD* standard deviation, *BMI* body mass index

Unless otherwise stated, data are reported per patient (n=117). Implant- or breast-level descriptors are reported per breast/implant (n=186)

Laterality was bilateral in 69 patients (59.0%), right-sided in 27 (23.1%), and left-sided in 21 (17.9%). Of 186 implants, 104 were Opticon (55.9%) and 82 Replicon (44.1%); 32 unilateral and 36 bilateral procedures used Opticon implants, while 16 unilateral and 33 bilateral procedures used Replicon implants. Twelve patients underwent SRM, nine of whom required FNG.

Most procedures were therapeutic (103 patients, 88.0%), with 14 prophylactic mastectomies (12.0%). Postoperative radiotherapy was administered to 58 patients (49.6%), and 9 (7.7%) had received radiotherapy preoperatively. Chemotherapy was given as neoadjuvant treatment in 52 patients (44.4%) and as adjuvant therapy in 19 (16.2%). Smoking was reported in 14 patients (12.0%), and comorbidities were present in 27 (23.1%). Genetic predisposition was identified in 24 patients (20.5%), including BRCA1, BRCA2, and other variants (Table 2).

**Table 2:** Surgical and oncologic characteristics

Variable	No	%
**Patient-level (N = 117)**		
*Implant side*		
• Bilateral	69	59.0
• Right	27	23.1
• Left	21	17.9
*Indication for surgery*		
• Therapeutic	103	88.0
• Prophylactic	14	12.0
*Radiotherapy*		
• Post-surgery	58	49.6
• Pre-surgery	9	7.7
• None	50	42.7
*Chemotherapy*		
• Neoadjuvant	52	44.4
• Adjuvant	19	16.2
• None	46	39.3
Skin-reducing mastectomy	12	10.3
Comorbidity	27	23.1
Smoking	14	12.0
*Genetic predisposition*		
• BRCA1	14	12.0
• BRCA2	1	0.9
• Other	9	7.7
• None	93	79.5
*BCCT.core score*		
• Excellent	20	17.1
• Good	75	64.1
• Fair	19	16.2
• Poor	3	2.6
Any complication	34	29.1
**Implant-level (n = 186 breasts)**		
*Implant base (per breast)*		
• Opticon	104	55.9
• Replicon	82	44.1

Patient-level variables are presented for N = 117 patients. Implant base is reported per breast (n = 186); bilateral patients contribute two breasts

Percentages are calculated within each denominator

*BRCA* breast cancer susceptibility gene, *BCCT.core* Breast Cancer Conservation Treatment software

### Postoperative Complications

Overall, 34 patients (29.1%) experienced at least one complication (Table 3). Rippling or upper-pole visibility was most common (19 patients, 16.2%), followed by wound dehiscence (5, 4.3%), hematoma (2, 1.7%), and implant loss (4, 3.4%). Three implant losses occurred in bilateral reconstructions, each affecting a single breast; one occurred in a unilateral case. Flap necrosis was seen in one patient (0.9%). Two patients developed late capsular contracture (1.7%), and one infection was treated successfully without implant removal (0.9%). No seroma was detected.

**Table 3:** Postoperative complications

Complication type	n	%
None	83	70.9
Rippling/upper-pole visibility	19	16.2
Implant loss—infection	3	2.6
Implant loss—exposure	1	0.9
Skin flap necrosis (secondary healing)	1	0.9
Late capsular contracture	2	1.7
Wound dehiscence (sutured closure)	5	4.3
Hematoma (evacuation)	2	1.7
Infection (antibiotics; no implant loss)	1	0.9

Incidence and distribution of postoperative complications. Each patient counted once per complication type

**Fig. 1:**
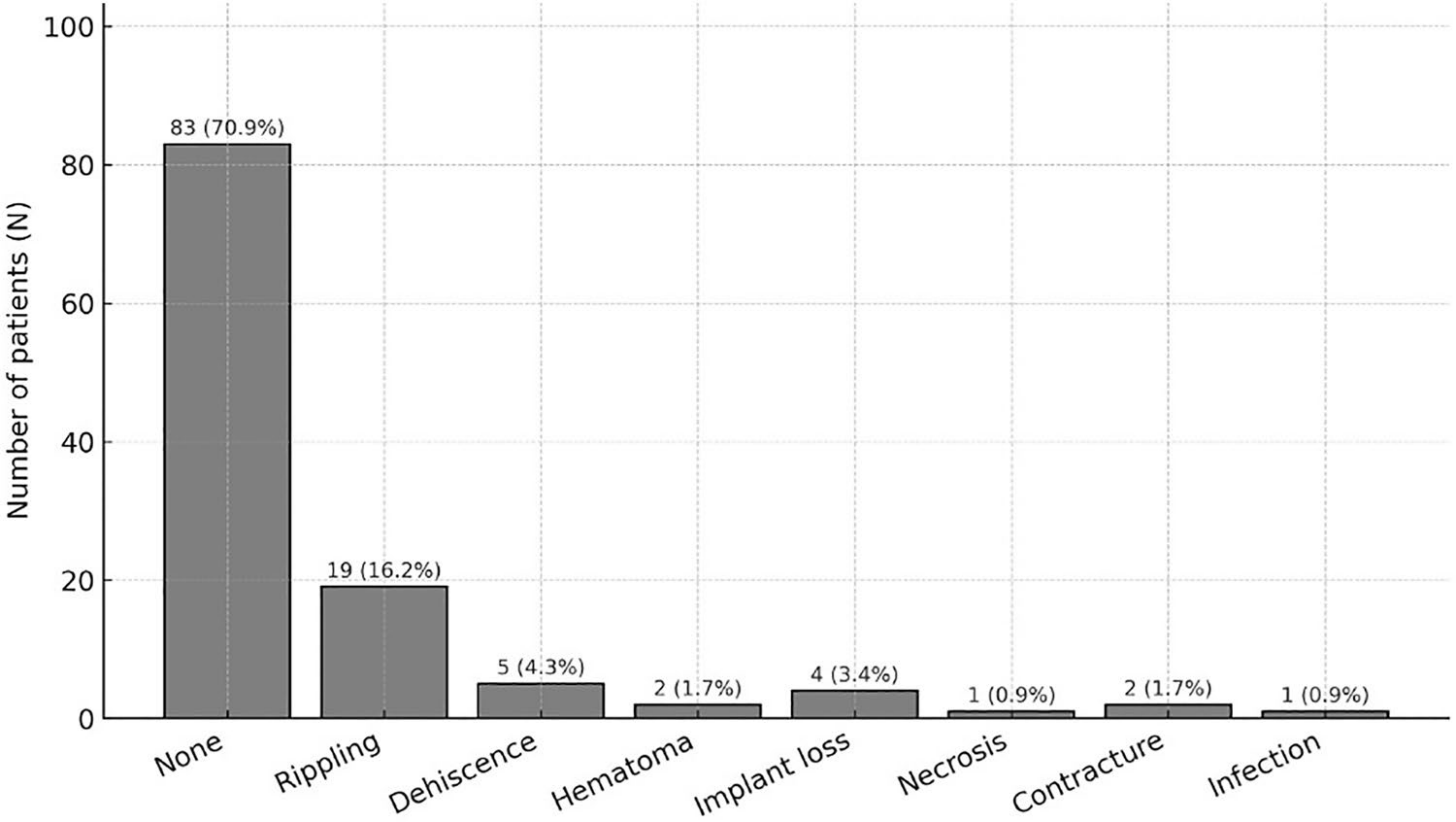
Distribution of postoperative complications. Incidence of postoperative complications among 117 patients, including rippling/upper-pole visibility, wound dehiscence, hematoma, implant loss, flap necrosis, infection, and capsular contracture

No implant malposition or rotation was noted. Clinical examinations focused on upper-pole shift, inferolateral displacement, and rotational asymmetry; imaging was reserved for equivocal findings but ultimately not required. At a median follow-up of 19 months, 70.9% remained complication-free. The distribution of complications is shown in Fig. 1.

### Factors Associated with Complications

On univariate analysis, patients with complications had higher BMI values (25.7 vs 24.1 kg/m^2^, *p *= 0.044) and received larger implants (419.9 vs 380.4 mL, *p* = 0.019). Complications were also more common in those undergoing SRM (53.3% vs 25.5%, *p* = 0.035). Smokers showed a higher—but nonsignificant—risk (50.0% vs 26.2%, *p* = 0.112). Radiotherapy, chemotherapy, comorbidity, and genetic predisposition showed no significant associations.

In the multivariable model, none of the evaluated variables reached statistical significance (Table 4). Implant size displayed a weak, nonsignificant trend (OR 1.05 per 10 mL; *p* = 0.196), while BMI showed no independent effect (OR 1.04; *p* = 0.610). Smoking and SRM demonstrated similar nonsignificant tendencies. Radiotherapy, chemotherapy, comorbidities, and genetic predisposition were not associated with complication risk.

**Table 4:** Univariate and multivariable logistic regression for predictors of any postoperative complication

Variable	Univariate *p* value	Adjusted OR	95% CI	Multivariable *p* value
Age (per year)	0.994	0.96	0.91 – 1.02	0.216
BMI (kg/m^2^)	0.044	1.04	0.89 – 1.23	0.610
Implant size (per 10 mL)	0.019	1.05	0.98 – 1.12	0.196
Smoking (Yes vs No)	0.112	2.77	0.77 – 9.94	0.118
Skin-reducing mastectomy	0.035	2.10	0.58 – 7.57	0.255
Radiotherapy (0/1/2)	0.187	1.35	0.68 – 2.69	0.391
Chemotherapy (0/1/2)	0.898	0.89	0.55 – 1.46	0.659
Comorbidity (Yes vs No)	0.752	1.24	0.39 – 3.95	0.717
Genetic predisposition	0.378	0.91	0.55 – 1.53	0.735

Univariate *p* values and adjusted odds ratios (OR) with 95% confidence intervals (CI) are shown for factors associated with postoperative complications (present vs absent). Because the total number of complication events was limited (*n* = 34), the multivariable model is considered exploratory, and estimates should be interpreted cautiously due to a low events-per-variable ratio

*OR* odds ratio, *CI* confidence interval, *BMI* body mass index, *RT* radiotherapy

Model calibration was acceptable (Hosmer–Lemeshow *χ*^2^ = 12.5, *p* = 0.13), though overall explanatory power was modest (Nagelkerke *R*^2 ^≈ 0.15).

### Cosmetic Outcomes (BCCT.core)

**Fig. 2:**
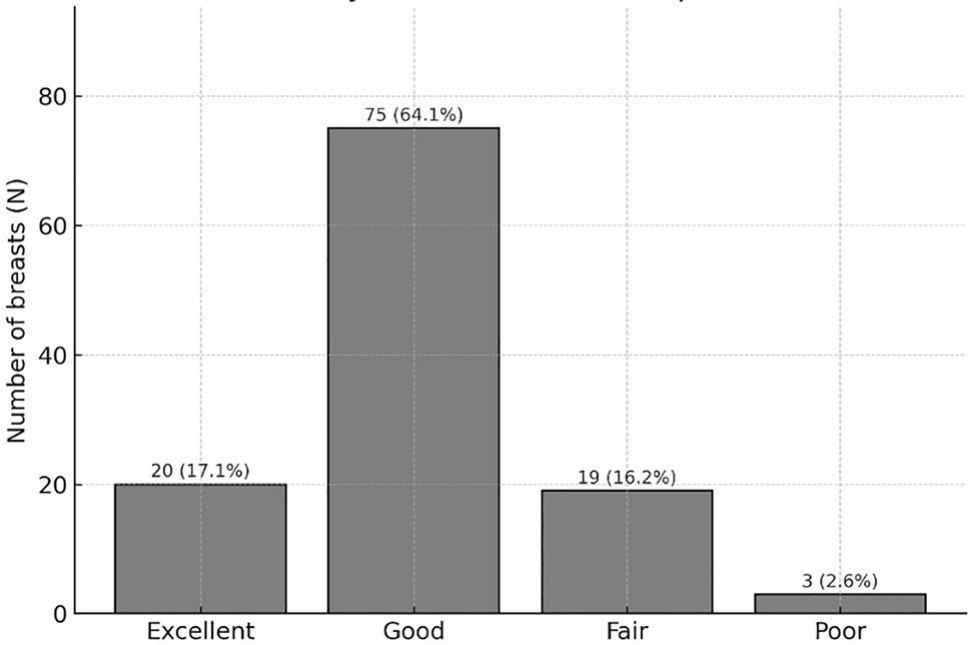
Cosmetic outcomes assessed by BCCT.core (n = 117). Distribution of BCCT.core aesthetic categories (Excellent, Good, Fair, Poor) among 117 patients. *BCCT.core* Breast Cancer Conservation Treatment software

BCCT.core evaluation showed that most reconstructions achieved favorable cosmetic results. Overall, 20 patients (17.1%) were rated excellent, 75 (64.1%) good, 19 (16.2%) fair, and 3 (2.6%) poor (Fig. 2).

Binary logistic regression showed that complications greatly reduced the chance of a favorable BCCT.core score (OR 0.21; 95% CI 0.07–0.70; *p* = 0.010), equivalent to a 4.8-fold higher probability of a good/excellent result in patients without complications. Increasing age was similarly associated with lower odds of a favorable outcome (OR 0.91 per year; 95% CI 0.84–0.99; *p* = 0.026). Larger implant volume showed a borderline trend toward poorer scores (OR 0.90 per 10 mL; 95% CI 0.82–1.00; *p* = 0.105), and radiotherapy demonstrated a comparable borderline tendency (OR 0.28; 95% CI 0.07–1.05; *p* = 0.059). Bilateral reconstructions displayed slightly higher descriptive BCCT.core scores than unilateral cases, although laterality was not included in the regression model (Table 5).

**Table 5:** Binary logistic regression for favorable BCCT.core score (good/excellent vs fair/poor)

Predictor	OR	95% CI	*p* value
Smoking (yes vs no)	4.80	0.67–34.32	0.118
Radiotherapy (yes vs no)	0.28	0.07–1.05	0.059
Skin-reducing mastectomy (yes vs no)	0.84	0.17–4.25	0.837
Comorbidity (yes vs no)	0.70	0.18–2.74	0.604
Complication (yes vs no)	0.21	0.07–0.70	0.010
Age (years)	0.91	0.84–0.99	0.026
BMI (kg/m^2^)	1.04	0.86–1.27	0.663
Implant size (per 10 mL)	0.90	0.82–1.00	0.105

Predictors of favorable cosmetic outcome according to BCCT.core. Odds ratios (OR) and 95% confidence intervals (CI) are shown. Implant size presented per 10 mL increase

*OR* odds ratio, *CI* confidence interval, *RT* radiotherapy, *BMI* body mass index, *BCCT.core* Breast Cancer Conservation Treatment software

In the ordinal logistic regression (coded excellent→poor), OR < 1 indicated lower odds of shifting into a worse aesthetic category; inverse ORs were used for clinical interpretation. Increasing age remained independently associated with poorer BCCT.core categories (inverse OR ≈ 1.06; *p* = 0.022), and postoperative complications again showed a strong adverse effect (inverse OR ≈ 4.0; *p* = 0.004). BMI, implant size, radiotherapy, smoking, SRM, and comorbidity were not significant predictors. Model fit was acceptable (Nagelkerke *R*^2 ^≈ 0.40) (Table 6).

**Table 6:** Ordinal logistic regression for BCCT.core scores (Excellent→Poor)

Predictor	OR	95% CI (low)	95% CI (high)	*p* value
Age (per year)	0.939	0.889	0.991	0.022
BMI (per kg/m^2^)	0.963	0.826	1.124	0.635
Implant size (per 10 mL)	0.959	0.901	1.021	0.194
Smoking (yes vs no)	2.083	0.620	6.999	0.235
Radiotherapy (0 none/1 post/2 pre)*	0.731	0.392	1.363	0.324
Skin-reducing mastectomy (yes vs no)	1.216	0.331	4.464	0.769
Any complication (yes vs no)	0.248	0.095	0.645	0.004
Comorbidity (yes vs no)	0.834	0.277	2.509	0.747

Outcome coded Excellent→Poor (1→4). OR<1 reflects reduced odds of shifting into poorer categories; inverse ORs (1/OR) were considered for clinical interpretation. Implant size per 10 mL. *OR* odds ratio, *CI* confidence interval, *RT* radiotherapy, *BMI* body mass index

Kruskal–Wallis analysis supported these findings: Age, BMI, and implant volume demonstrated stepwise gradients across BCCT.core categories, with median values progressively shifting from excellent to poor (age: 38.5→43→47→52 years, *p* = 0.008; BMI: 22.6→24.2→24.9→25.0 kg/m^2^, *p* = 0.035; implant volume: 357.5→390→435→400 mL, *p* = 0.007), indicating that younger age, lower BMI, and smaller implants consistently aligned with better aesthetic outcomes (Table 7).

**Table 7:** Kruskal–Wallis analysis of continuous variables across BCCT.core categories

Variable	Poor (n=3)	Fair (n=19)	Good (n=75)	Excellent (n=20)
Age (years)	52.0	47.0	43.0	38.5 (*p*=0.008)
BMI (kg/m^2^)	25.0	24.9	24.2	22.6 (*p*=0.035)
Implant volume (mL, median)	400.0	435.0	390.0	357.5 (*p*=0.007)
Implant volume (per 10 mL)	40.0	43.5	39.0	35.8 (*p*=0.007)

Median values for age, BMI, and implant volume across BCCT.core aesthetic categories (Poor, Fair, Good, Excellent). *P* values calculated using the Kruskal–Wallis test

*BMI* body mass index, *BCCT.core* Breast Cancer Conservation Treatment software

**Fig. 3:**
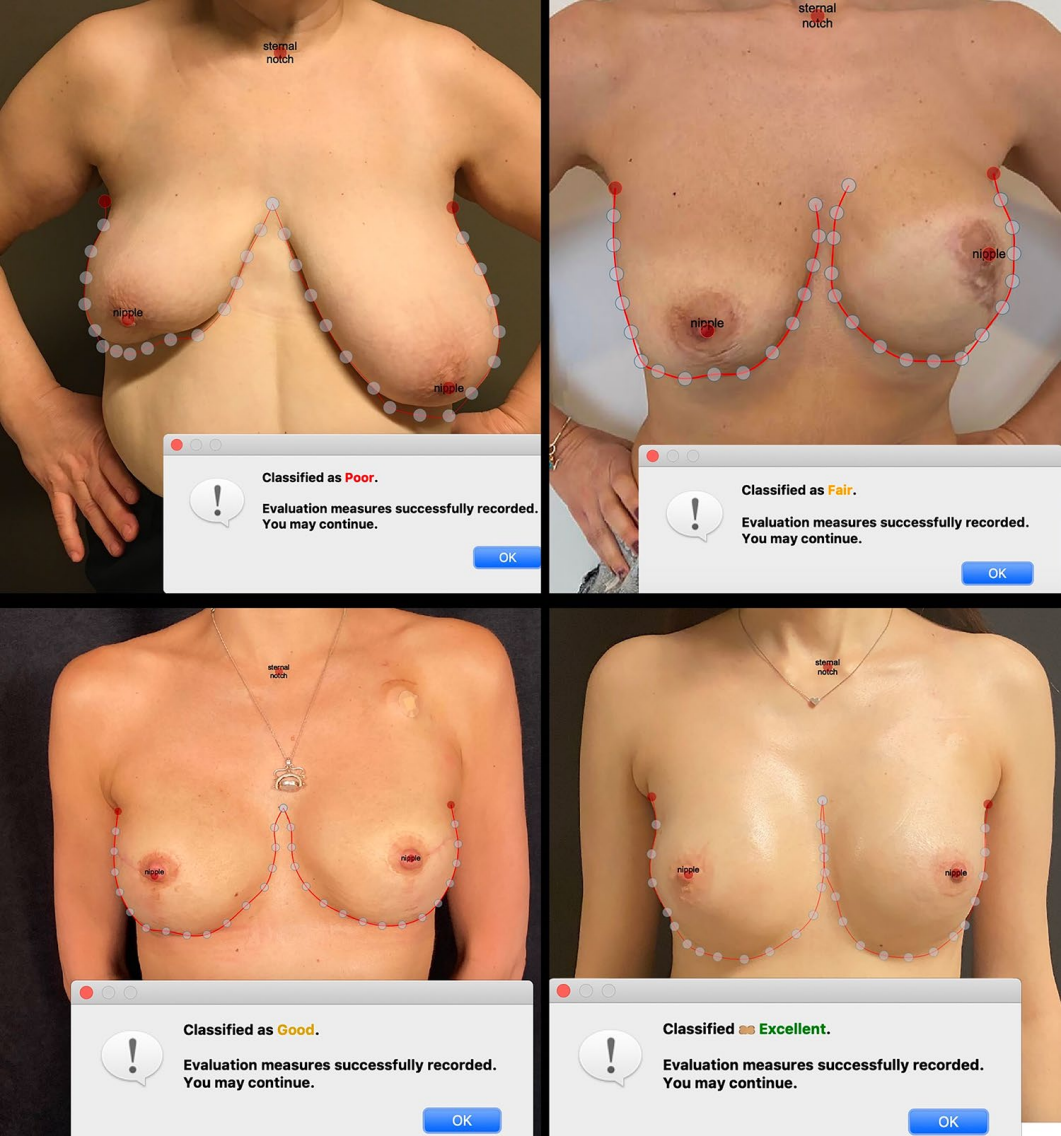
Representative clinical examples corresponding to BCCT.core aesthetic categories

**Fig. 4:**
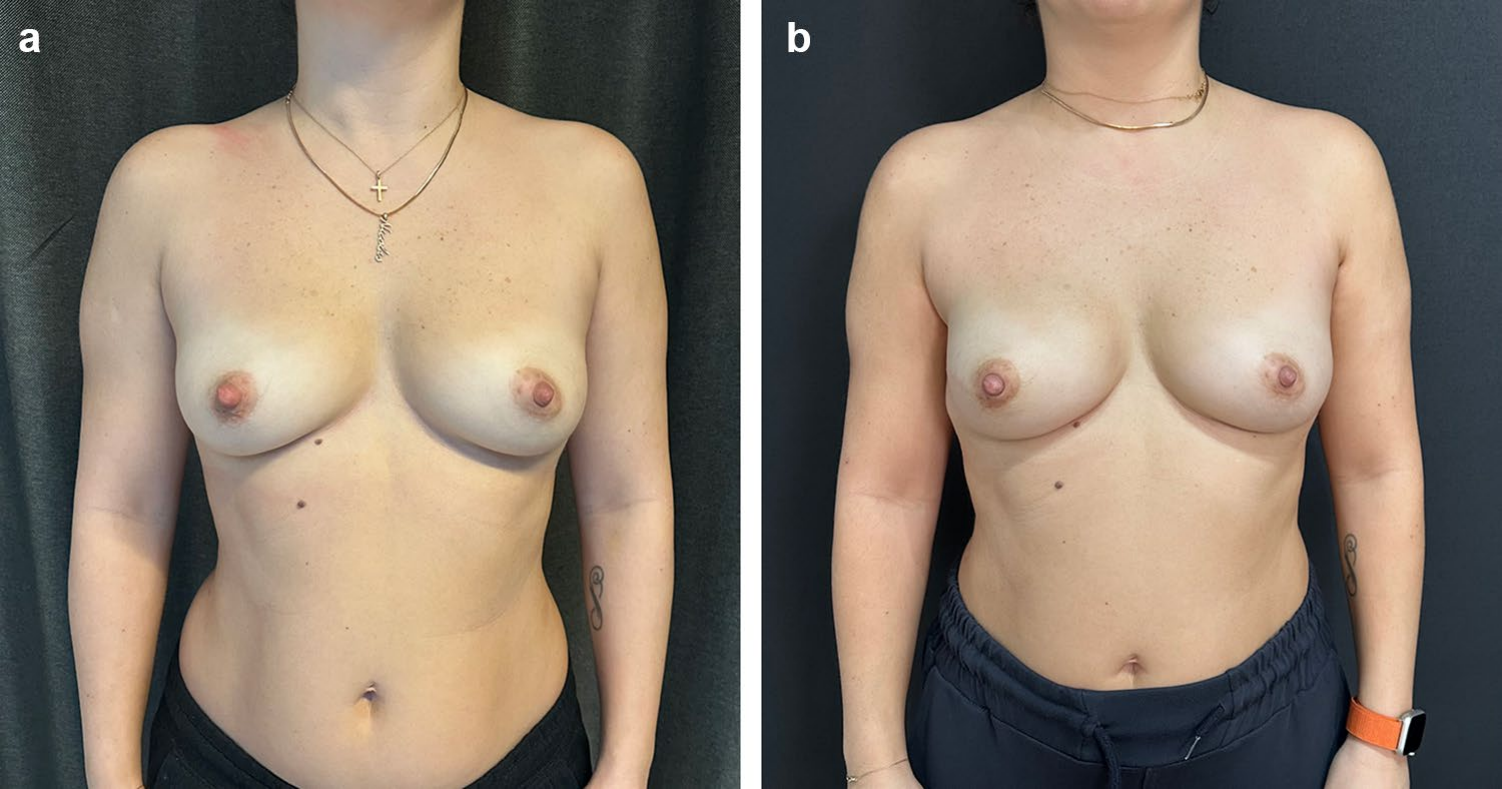
**a** Preoperative view demonstrating right breast carcinoma. BMI 23.4. Age: 36. **b** Twelve-month postoperative appearance following nipple-sparing mastectomy and prepectoral implant-based reconstruction. No radiotherapy or chemotherapy was administered. Reconstruction performed with an Opticon 45-265 cc; BCCT.core aesthetic score: Good

**Fig. 5:**
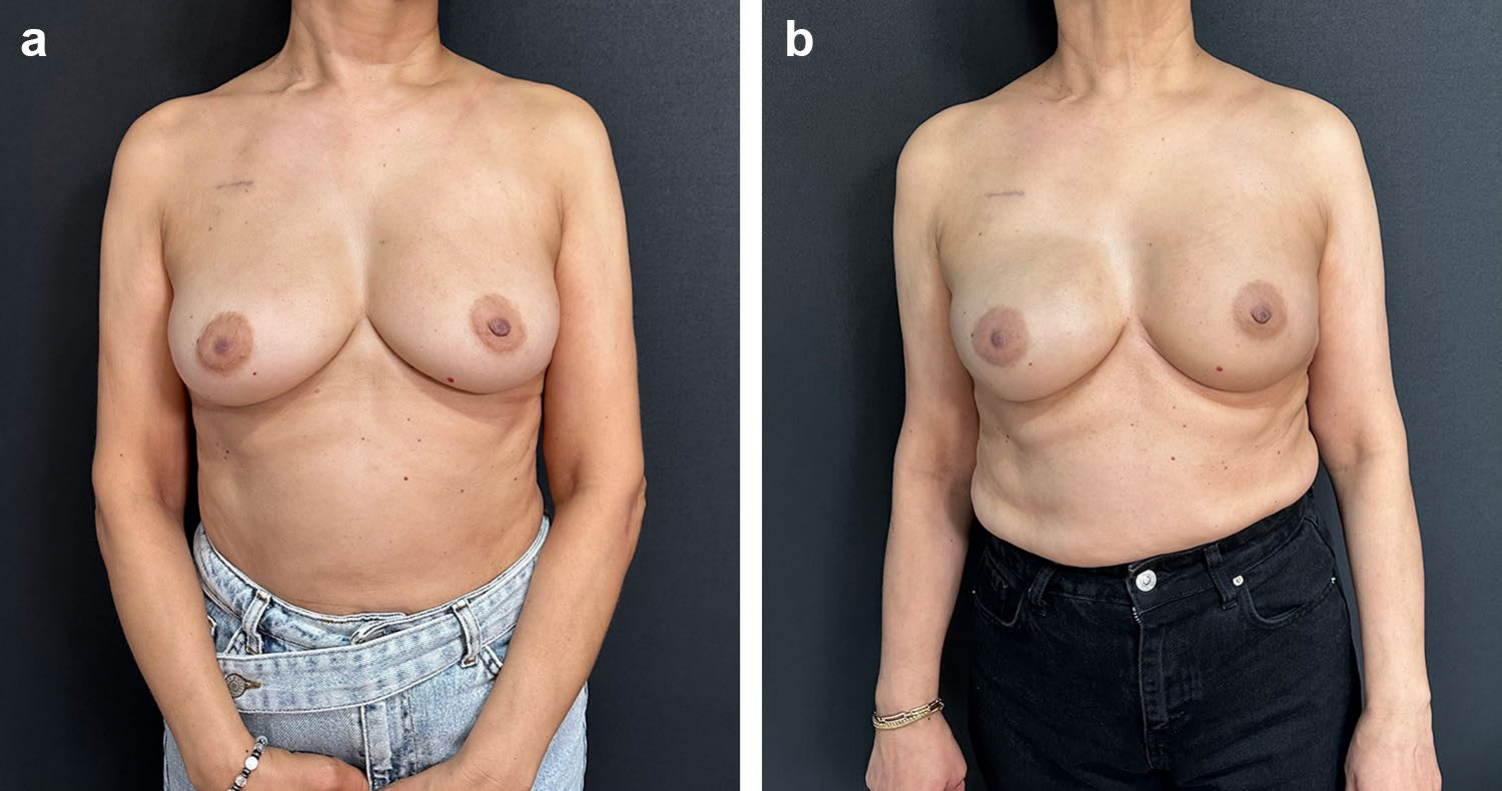
**a** Preoperative view demonstrating left breast carcinoma. BMI 22.2. Age: 51. **b** Six-month postoperative result following nipple-sparing mastectomy, neoadjuvant chemotherapy, and postoperative radiotherapy. Reconstruction performed with an Opticon 46-335cc implant; BMI 22.2. BCCT.core aesthetic score: Excellent

**Fig. 6:**
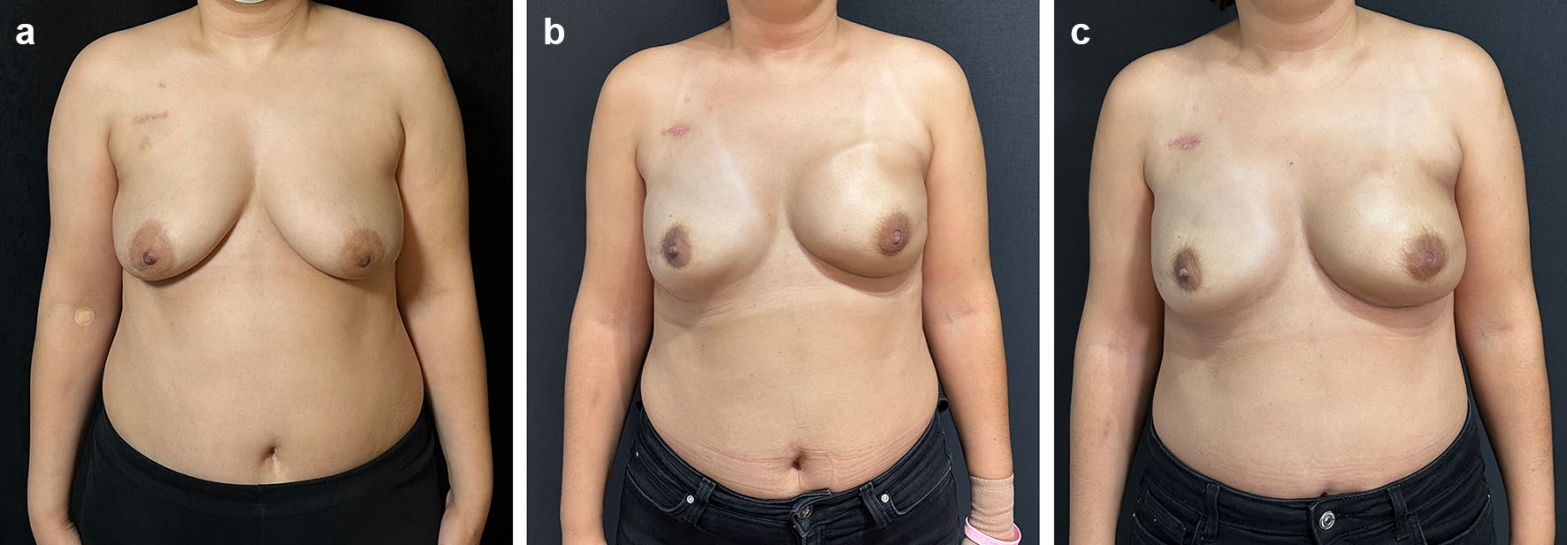
**a** Preoperative view demonstrating left breast carcinoma. BMI 29.13. Age: 31. **b** Nine-month postoperative appearance following bilateral skin-sparing mastectomies and prepectoral reconstruction after neoadjuvant chemotherapy and radiotherapy. Reconstruction performed with Replicon 35-390cc implants. Upper pole edge visibility is noted in the left breast. **c** Six weeks after a 200 cc fat injection

Figure 3 illustrates representative BCCT.core classifications, demonstrating the typical features of poor, fair, good, and excellent results. Figures 4, 5 and 6 provide representative clinical examples, showing preoperative presentation and postoperative aesthetic outcomes in unilateral right-sided reconstruction (Fig. 4a, b), left-sided neoadjuvant therapy reconstruction (Fig. 5a, b), and bilateral reconstruction including secondary fat injection (Fig. 6a–c).

## Discussion

Prepectoral implant-based breast reconstruction has gained broad acceptance as an alternative to subpectoral placement because it avoids muscle dissection, reduces postoperative pain, and eliminates animation deformity. These benefits must be balanced against concerns about long-term safety, especially in smokers, high-BMI patients, and those receiving radiotherapy [16].

Within this context, PU-coated implants have re-emerged as a strategy to enhance positional stability and modify the soft-tissue response observed with smooth or textured devices. Their microporous foam surface promotes tissue ingrowth, limits micro-motion, and has been associated with reduced malposition and lower capsular contracture rates in contemporary series [17–19]. This adhesive “Velcro effect” is particularly advantageous in the prepectoral plane, where implant stability is determined largely by mastectomy flap quality [20].

The overall complication rate in this study was 29.1%. Rippling was the most common minor issue, implant loss remained low at 3.4%, and capsular contracture occurred in 1.7% of reconstructions—all in irradiated breasts—consistent with Pompei et al. [21] and underscoring the impact of radiotherapy across implant types [22–24].

The very low infection rate observed in this series may reflect careful patient selection for direct-to-implant reconstruction, limited pocket dissection, and a postoperative protocol with continued antibiotic coverage until drain removal. The absence of ADM, together with the rapid tissue adhesion of the polyurethane surface, may also reduce dead space, although this observation should be interpreted cautiously given the retrospective design. This finding is supported by a systematic review and meta-analysis reporting lower infection rates in implant-based breast reconstruction when postoperative antibiotics were administered, although the optimal duration remains debated [25].

Patient selection played a role: higher BMI and larger implants correlated with complications in univariate analysis, matching reports linking body habitus and implant size to increased tension and poorer perfusion [26]. SRM was similarly associated with higher risk, reflecting the intrinsic challenges of the procedure, while smoking followed the expected impairment in wound healing [27].

In skin-reducing mastectomy cases, free nipple–areola grafting was preferred to reduce the risk of ischemic complications in patients with marked ptosis and long skin flaps. Although immediate nipple preservation using local or superiorly based flaps has been described [28, 29], these techniques require thicker and more reliably perfused flaps. In our practice, NAC grafting was considered a safer and more reproducible option in selected high-risk cases, particularly when combined with prepectoral implant placement. A similar approach has been reported in patients undergoing skin-reducing mastectomy with immediate reconstruction, where free nipple–areola complex grafting was used with acceptable complication rates and reliable graft survival [30].

None of these factors remained significant after adjustment, likely due to the limited number of events and the low events-per-variable ratio in the multivariable model. Some trends—especially smoking and implant size—remained clinically relevant but underpowered. Flap thickness, a key determinant of safe prepectoral placement, was assessed clinically rather than objectively [31].

One published series has reported favorable outcomes with prepectoral reconstruction supported by acellular dermal matrices, showing acceptable complication rates and good aesthetic results in selected patients [32]. In contrast, our series relied exclusively on polyurethane-coated implants without ADM, aiming to achieve implant stability through tissue adhesion rather than additional reinforcement. The low rates of implant loss and capsular contracture observed in our cohort are comparable to those reported in ADM-assisted prepectoral reconstruction, while avoiding the added cost and foreign material burden associated with ADM use. Direct comparison is limited by differences in patient selection and study design, but our findings suggest that PU-coated implants may represent a valid ADM-free alternative in appropriately selected cases.

Aesthetic outcomes were generally favorable; more than 80% of reconstructions achieved excellent or good BCCT.core scores. Younger age, lower BMI, and smaller implants correlated with better categories in univariate testing, while postoperative complications consistently shifted results toward fair/poor. Ordinal regression confirmed that older age and complications were the main predictors of poorer BCCT.core outcomes. Radiotherapy and implant size showed mild, nonsignificant trends; overall model performance (Nagelkerke *R*^2 ^≈ 0.40) was comparable to other PU-based series.

As expected, bilateral reconstructions showed more consistent symmetry than unilateral cases, reflecting inherent advantages rather than an implant-specific effect. In unilateral cases, the adhesive characteristics of the PU surface support stable positioning and long-term contour [33]. In unilateral reconstruction, the strong tissue adhesion associated with the polyurethane surface may represent both an advantage and a limitation. While early positional stability and contour control are improved, long-term asymmetry may theoretically become more evident as the native breast undergoes physiological aging and ptosis. This potential trade-off should be considered during patient counseling and highlights the need for longer follow-up to better define the long-term aesthetic behavior of PU-coated implants in unilateral cases. PU implants require some adjustments compared with smooth devices, but the learning curve is short, and outcomes improve with experience [34]. No cases of BIA-ALCL were observed. Current evidence suggests that BIA-ALCL remains rare and mainly linked to textured devices, but precise risk estimates for PU implants are still uncertain because exposure data and reporting vary across countries. Continued patient counseling and surveillance remain important [35–38].

Compared with previously published series, this study provides one of the larger single-center experiences focusing exclusively on polyurethane-coated implants in the prepectoral setting and incorporates an objective, software-based aesthetic evaluation. The combination of complication analysis with BCCT.core assessment offers complementary insight into both clinical safety and aesthetic performance, particularly in an ADM-free reconstructive strategy. These aspects may help refine patient selection and counseling in routine clinical practice.

## Limitations

This study has several limitations. Its retrospective, single-center design introduces selection bias and limits generalizability. Selection bias is also possible because smokers, patients with higher BMI, or those expected to receive radiotherapy were not excluded but were counseled and treated selectively. Microthane implants are not available in the USA, and regional differences in implant availability restrict broader applicability. The follow-up period is relatively short for assessing late complications such as capsular contracture or long-term oncologic safety. Complication timing was not recorded in a standardized manner, precluding time-to-event analysis.

Some subgroups were small—such as prophylactic mastectomy (*n* = 14), BRCA2 mutation carriers (n=1), and patients receiving preoperative radiotherapy (*n* = 9)—leading to wide confidence intervals and occasional non-estimable coefficients. The modest number of complication events also limits the statistical power of multivariable models, rendering those findings exploratory.

Flap thickness was evaluated only clinically and not measured objectively, although it is a key determinant of prepectoral safety and implant visibility. This reflects routine clinical practice but limits a more precise risk stratification. Aesthetic analysis relied solely on BCCT.core, which does not substitute for patient-reported outcomes; tools such as BREAST-Q should be integrated in future work. Finally, the lack of a comparator cohort using smooth or ADM-supported implants restricts direct benchmarking.

Larger prospective studies with longer follow-up and inclusion of patient-reported outcomes are needed to validate and expand upon these findings. Nevertheless, these limitations do not invalidate the main conclusions of the study, as implant loss and major complications usually occur early after reconstruction, implant positioning remained stable during follow-up, and the objective aesthetic analysis showed consistent associations with age and postoperative complications.

## Conclusion

Prepectoral breast reconstruction with PU-coated implants provided low rates of implant loss and capsular contracture, together with generally favorable BCCT.core scores. Complications and increasing age were the main factors associated with poorer aesthetic outcomes. PU-coated implants showed stable positioning in the prepectoral plane.
